# A New Disinfection Approach Using a Chitosan-Based Endodontic Irrigant

**DOI:** 10.3390/ma18245552

**Published:** 2025-12-10

**Authors:** Alejandra Itzel Lopez-Flores, Ulises Velazquez-Enriquez, Rogelio Jose Scougall-Vilchis, Laura Susana Acosta-Torres, Laura Emma Rodriguez-Vilchis, Rosalía Contreras-Bulnes, Paloma Netzayeli Serrano-Diaz, Rene Garcia-Contreras

**Affiliations:** 1 Facultad de Odontología, Universidad Autónoma del Estado de México, Toluca 50130, MX, Mexico; alopezf020@alumno.uaemex.mx; 2 Dental Advanced Research Center, Facultad de Odontología, Universidad Autónoma del Estado de México, Toluca 50130, MX, Mexico; rscougallv@uaemex.mx (R.J.S.-V.); lerodriguezv@uaemex.mx (L.E.R.-V.); rcontrerasb@uaemex.mx (R.C.-B.); 3 Biomaterials and Nanostructures Department, Escuela Nacional de Estudios Superiores, Universidad Nacional Autónoma de México, León 37684, GTO, Mexico; lacosta@enes.unam.mx (L.S.A.-T.); pserranod@enes.unam.mx (P.N.S.-D.); rgarciac@enes.unam.mx (R.G.-C.)

**Keywords:** root canal irrigants, chitosan, irrigators, antibiotics, antibacterial effect

## Abstract

The use of chitosan nanoparticles (CH-NPs) loaded with antibiotics as irrigants in endodontics offers a unique combination, demonstrating effective antibacterial activity and low toxicity. Therefore, the aim of this study was to develop chitosan nanoparticles loaded with metronidazole, ciprofloxacin, and minocycline for use as endodontic irrigants to improve antibacterial activity against Enterococcus faecalis and to evaluate their cytotoxicity in human dental pulp stem cells (hDPSCs). *Methods:* The CH-NPs loaded with antibiotics were synthesized and analyzed using Fourier Transform Infrared Spectroscopy (FTIR) and Ultraviolet-visible spectroscopy (UV-Vis). Agar diffusion and microdilution assays were performed to determine the minimum inhibitory concentration (MIC), and a cytotoxicity assay was conducted to establish the median cytotoxic dose. *Results:* Peaks corresponding to the functional groups of the chitosan-antibiotic complex’s chemical structure were identified. A one-way ANOVA (*p* ≤ 0.05) with Tukey’s post hoc test was used to analyze the antibacterial effect. CH-NPs-ciprofloxacin showed the greatest antibacterial activity against *E. faecalis* in both agar diffusion and microdilution assays. CH-NPs-metronidazole demonstrated lower cytotoxicity against hDPSCs. CH-NPs-minocycline showed superior antibacterial effects compared to plain chitosan in microdilution assays, although they exhibited greater cytotoxicity. Conclusions: The ongoing search for an irrigating solution with effective antibacterial properties and low cytotoxicity could transform traditional techniques. However, this field is still developing and underexplored. It is essential to reevaluate decisions about irrigation solutions, as evidence on the use of chitosan nanoparticles with antibiotics is limited. This study provides valuable data for endodontics and is a crucial step for future research.

## 1. Introduction

Dental caries is a complex disease whose development is mediated by the presence of bacterial biofilms. These biofilms cause demineralization of the tooth’s hard tissues, as well as destruction of the soft tissue [1]. Treatment of caries typically involves the removal of the affected tissue [2]; however, some microorganisms can persist and form an endodontic biofilm. The formation of this biofilm is influenced by the presence of organic debris, which acts as a substrate and creates a favorable environment for bacterial growth [3,4]. Inside the root canals, bacteria can be found both as free-floating cells (planktonic) and as aggregates that adhere to the canal walls. Endodontic biofilms represent a unique model of bacterial growth, in which cells form dynamic communities on a substrate, embedded within an extracellular matrix [5].

Understanding the dynamics of endodontic biofilms is crucial for developing effective treatments for dental caries. Endodontic treatment involves the removal of the pulp tissue, disinfection of the root canal with irrigants, application of intracanal medications, and sealing of the canal with an inert material [6,7,8]. While it has an overall success rate of approximately 85% to 90% in cases of irreversible pulpitis and 66% in retreatment procedures, challenges remain, as some bacteria can adapt to nutrient-poor environments, potentially leading to apical lesions [7]. *Enterococcus faecalis* (*E. faecalis*), a highly pathogenic Gram-positive bacterium, is of particular concern in endodontic treatments due to its association with the failure rate of these procedures. This bacterium has the ability to adhere to non-living surfaces, invade dentinal tubules, hinder the penetration of some antibacterial agents, and colonize tissues. Its high pathogenicity justifies the need to develop effective treatment strategies [9,10,11,12,13,14].

Root canal treatment constitutes a vital component in the eradication of endodontic biofilm; it involves the chemical and mechanical debridement of pulp tissue, followed by the disinfection of the root canals through the application of endodontic irrigants and the obturation with an inert material [15]. Endodontic irrigants are chemical substances used to disinfect the root canals during mechanical instrumentation, playing a crucial role in endodontic treatment. However, these chemically active solutions used for treating biofilms in the root canal system have certain limitations, such as the complex morphology of the root canals, adverse effects on dentin that alter its structural and mechanical properties, and the risk of extrusion through the apical foramen, which can cause tissue damage and pain-all of which represent significant challenges [14,16]. The most commonly utilized irrigants are Chlorhexidine (CHX) and Sodium hypochlorite (NaOCl). NaOCl is an irrigant that dissolves organic and inorganic tissue and causes the hydrolysis and oxidation of some cellular proteins. Nevertheless, its cytotoxic effects are a concern, as they may be triggered by the inadvertent extrusion of the irrigant into the periapical tissues, presenting a significant clinical risk and inducing severe pain and necrosis of the surrounding tissues. CHX is considered a safer irrigant for endodontic applications; however, certain limitations have been documented regarding its efficacy in removing endodontic biofilm [17].

For this reason, complementary strategies have been implemented to ensure the complete removal of biofilm by administering medications directly into the root canal, which are indicated for the treatment of periapical lesions, root resorption, apexogenesis, and revascularization [18,19]. Some antibiotics used include metronidazole, ciprofloxacin, and minocycline. However, these antibiotics exhibit high toxicity when applied directly to dental tissues, and minocycline can cause significant tooth discoloration [18,20]. Chitosan (CH) exhibits a cellulose-like structure comprising two types of repeating units: N-acetyl-D-glucosamine and D-glucosamine, connected through β-glycosidic bonds. It is regarded as a natural polysaccharide that has been extensively studied in the field of endodontics, owing to its notable anti-inflammatory, antifungal, and antibacterial properties, as well as its low toxicity, high biocompatibility, and biodegradability within dentin root canals. Chitosan-based nanocomposites demonstrate considerable versatility in their manipulation, as they can be formulated as nanoparticles or scaffolds of various shapes and sizes, thereby facilitating cell adhesion and differentiation [21,22,23,24]. The utilization of chitosan nanoparticles (CH-NPs) in endodontic root canal therapy signifies a noteworthy advancement, given that their mechanism of action hinges on electrostatic interactions between the positively charged CH-NPs and the negatively charged surfaces of bacterial species, thereby increasing cell wall permeability and ultimately causing cell death. It is therefore pertinent to emphasize that the future trajectory of endodontics is increasingly oriented towards integrating nanotechnology, a field that holds considerable promise in dentistry [17,25]. As an adjunct to root canal therapy, the employment and prescription of broad-spectrum antibiotics administered orally for systemic effects have also been proposed; however, their efficacy is confined to pathologies that extend beyond the root canals, necessitating careful evaluation of their prescription [17]. Currently, studies have begun to report on the application of nanoparticles-loaded PLGA microcapsules for enhanced encapsulation of chitosan, metronidazole, ciprofloxacin, minocycline, and toluidine blue, showing an antibacterial effect on *E. faecalis*, demonstrating significant reductions in colony re-encounter and low cytotoxicity [25]. However, the scientific evidence on the use of chitosan as an endodontic irrigant, particularly when loaded with metronidazole, ciprofloxacin, or minocycline to combat bacteria responsible for endodontic treatment failure, remains limited. Therefore, the aim of this research is to develop chitosan nanoparticles loaded with metronidazole, ciprofloxacin, and minocycline, which can be used as an endodontic irrigant to enhance their antibacterial effect against *E. faecalis*, and to evaluate their cytotoxicity on human dental pulp stem cells (hDPSCs).

## 2. Materials and Methods

### 2.1. Preparation of Chitosan Nanoparticles (CH-NPs)

Using the ionic gelation method described by Rizeq et al. [26], chitosan nanoparticles (CH-NPs) were synthesized Initially, First 1 g of low-molecular-weight chitosan (Sigma Aldrich, St. Louis, MO, USA) was dissolved in a 1% acetic acid solution (Sigma Aldrich, St. Louis, MO, USA) and stirred on a magnetic stirrer (IKA Works, Wilmington, DE, USA) at 350 rpm for 24 h. The synthesis was conducted twice: once at room temperature (W/OT) and once at 60 °C (W/T). Next, we prepared a 10 mL/5 mg TPP solution (sodium tripolyphosphate, Sigma-Aldrich, St. Louis, MO, USA) that interacts electrostatically with the amino groups of chitosan to form stable, spherical nanoparticles in sterile, triple-distilled water (Laboratorios Pisa, Mexico City, Mexico) at pH 4.7, adjusted with NaOH. This process is used to encapsulate and release antibiotics in a controlled manner [26]. Each antibiotic, metronidazole, ciprofloxacin, and minocycline (Sigma Aldrich, St. Louis, MO, USA; CAS: 443 48; CAS: 443-48-1; CAS: 13614-98-7) was dissolved in 10 mL of sterile tri-distilled water at a concentration of 100 mg. These solutions were filtered through a 0.45 µm membrane, then through a 0.2 µm Millipore membrane (Sigma-Aldrich, St. Louis, MO, USA), and stirred for 40 min at 350 rpm [27,28].

### 2.2. Characterization Techniques

The CH-NPs were analyzed using FTIR to identify and compare the functional groups of chitosan, metronidazole, ciprofloxacin, and minocycline in a spectrum range of 400 to 4500 cm^−1^ and Ultraviolet-visible spectroscopy (UV-Vis, Multiskan Go, Thermo Fisher Scientific, Waltham, MA, USA) was used at a wavelength range of 100–500 nm with a 1 nm resolution [28].

### 2.3. Inhibition of Growth

Agar diffusion and microdilution assays were performed according to the guidelines outlined in ISO 9001:2015 [29], M100 [30], M02 [31] and M07 [32], by the Clinical and Laboratory Standards Institute and NOM-210-SSA1-2014 [33], NOM-010-STPS-2014 [34].

#### 2.3.1. Agar Diffusion Assay

*E. faecalis* (clinical isolate) was cultured on Petri dishes (Thermo Scientific, Richardson, TX, USA) with 38 g of Mueller-Hinton agar (Sigma Aldrich Brand, Oxoid, Basingstoke, UK) dissolved in 1000 mL of sterile distilled water (Pisa Laboratories, Jalisco City, Mexico) using a magnetic stirrer (Ika Works, Wilmington, DE, USA) at 120 °C. 20 mL was poured into each Petri dish (Thermo Scientific, Richardson, TX, USA) to prepare the young culture by the streaking technique and incubated at 37 °C for 24 h. Four colonies were taken from the fresh culture, adjusting the solution to a turbidity standard of 0.5 on the McFarland scale corresponding to 1 × 10^4^ CFU/mL, in a 0.85% NaCl solution [35,36]. Once the solution was ready, 140 μL of *E. faecalis* was placed and evenly distributed throughout the dish using a sterile swab. Subsequently, five wells were made in each Petri dish. Antibiotic-loaded CH-NPs (100 μg/mL, 50 μg/mL, and 25 μg/mL) were synthesized in ratios of 1:1, 1:0.5, and 1:2, with and without temperature control during the synthesis. Twenty μL of each chitosan-drug solution was placed in each well and left in the incubator (Vortemp 1550, Labnet, Edison, NJ, USA) for 24 h. The inhibition zones were measured using a Vernier caliper. This assay was performed in triplicate (*n* = 90) [37,38].

#### 2.3.2. Microdilution Assay

To determine the minimum inhibitory concentration (MIC) of the *E. faecalis* strain using a CH broth solution containing antibiotics, a microdilution assay was performed using 96-well plates. The plates were divided into groups according to different concentrations of CH with antibiotics, ranging from 100 μg/mL to 3.12 μg/mL. Fresh culture broth was prepared, and 10 mL of Muller-Hinton broth (Sigma Aldrich Brand, Oxoid, Basingstoke, UK) was placed in a test tube. Three to four colonies from the fresh culture were transferred and suspended in the solution by shaking at 120 rpm for 16 h at 37 °C (overnight). The bacterial inoculum was diluted (1:1000) to obtain a final concentration of 1 to 1 × 10^4^ CFU/mL, and the solution was adjusted to a turbidity of 0.5 on the McFarland scale. Once prepared, 100 µL of the bacterial culture medium was added to each well. For the experimental groups, 100 µL of the CH solution with antibiotics was added, along with 100 µL of CHx as a positive control (FGM, Joinville, Santa Catarina, Brazil). Each well contained a total of 200 µL of solution. The plates were incubated in a shaking incubator at 37 °C for 24 h at 120 rpm (Vortemp 1550, Labnet, Edison, NJ, USA). The MIC was determined by turbidity, considering the concentration at which no viable cells were observed (absence of turbidity), in comparison to the negative control. The optical density was normalized, with the value of the negative control representing 100% bacterial growth [39,40].

#### 2.3.3. Bacterial Viability Quantification from the Microdilution Assay

The contents of the wells were aspirated, taking care not to disturb the sediment, and 100 µL of MTT (thiazolyl blue tetrazolium bromide, Sigma-Aldrich, St. Louis, MO, USA) at a concentration of 0.0002 g/mL was added. The mixture was incubated at 37 °C for 4 h. After this incubation period, the solution was aspirated again. Subsequently, 100 µL of dimethyl sulfoxide (DMSO, Karal, Guanajuato, Mexico) was added to each well to measure the optical density using UV-VIS spectroscopy at 570 nm. The data were obtained from three independent experiments performed in triplicate (*n* = 9) [39,40].

### 2.4. Cell Cultures

In accordance with ISO 10993-5:2009 [41], human dental pulp stem cells (hDPSCs) were obtained from the Interdisciplinary Laboratory for Research in Nanomaterials and Biomaterials at the León Unit of the National School of Higher Studies (ENES)—León Unit, UNAM. The cells were stored at −80 °C. The hDPSCs were inoculated onto culture plates containing minimal essential medium (MEM, Gibco, Carlsbad, CA, USA) supplemented with 1% glutamine (Sigma-Aldrich, St. Louis, MO, USA) and 1% penicillin/streptomycin (Sigma-Aldrich, St. Louis, MO, USA). The culture medium was changed every 2 days until cell confluence reached 80–90%, and the plates were incubated at 37 °C, 95% humidity, and 5% CO_2_ for 24 h. Subsequently, the hDPSCs were subcultured in 96-well plates (Becton Dickinson, Franklin Lakes, NJ, USA) at a concentration of 5 × 10^4^ cells/mL and incubated at 37 °C with 95% humidity and 5% CO_2_ for 24 h. For the experimental groups, 100 µL of the medium containing hDPSCs was mixed with 100 µL of CH solution, with or without antibiotics, at concentrations ranging from 100 µg/mL to 1.56 µg/mL, resulting in a total volume of 200 µL per well [40,42,43,44,45].

#### MTT Cytotoxicity Assay

The cytotoxic effects of CHNPs were determined by direct contact, with cell viability assessed using an MTT assay. After adding 100 µL of MTT, the samples were incubated for an additional 4 h to allow the formation of formazan crystals. These crystals were then dissolved with 100 µL of DMSO (Karal, Guanajuato, Mexico). Optical density readings were obtained at 570 nm using UV-Vis spectroscopy. Finally, the average cell cytotoxicity value (CC_50_) was determined. Cell viability was calculated using the following formula: cell viability (%) = (optical density of the experimental group/optical density of the control group) × 100. Data were obtained from three independent experiments performed in triplicate (*n* = 9) [40,42,43,44,45].

### 2.5. Statistical Analysis

Data were analyzed using mean and standard deviation, and a one-way ANOVA with Tukey’s multiple comparisons (*p ≤* 0.05). Prism 10 for Windows, version 10.2.3, 2024, was used.

## 3. Results

### 3.1. Characterization of the CH-NPs with FTIR and UV-Vis

**Figure 1:**
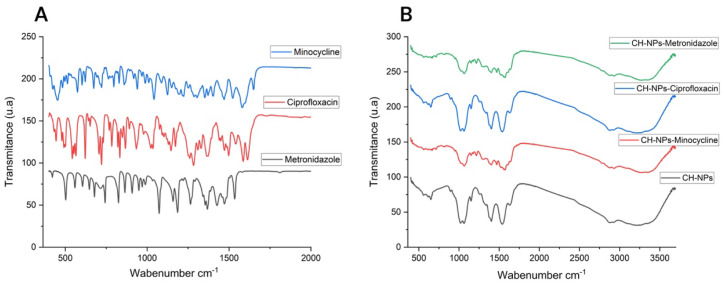
(**A**) FTIR spectra of Antibiotics, (**B**) FTIR spectra of CH-NPs and CH-NPs loaded with antibiotics.

The FTIR spectra of CH (Figure 1B) show characteristic peaks at 3325 cm^−1^ (O-H stretching), 2986 cm^−1^ (C-H stretching), 1467 cm^−1^ (C=O stretching), 1605 cm^−1^ (NH_2_; primary amine), and 1048 cm^−1^ (C-O stretching). Characteristic peaks attributable to the presence of antibiotics are also observed: at 1651 cm^−1^ (in-plane bending of NH_2_; secondary amine), 1350 cm^−1^ (CN stretching; primary amine), and 1577 cm^−1^ (NH_2_; primary amine) corresponding to minocycline (Figure 1A); at 1075 cm^−1^ (C-F; characteristic of halogenated organic molecules) corresponding to ciprofloxacin (Figure 1A); and at 1076 cm^−1^ and 1267 cm^−1^ corresponding to the asymmetric S=O stretching and C-O stretching of metronidazole (Figure 1A).

**Figure 2:**
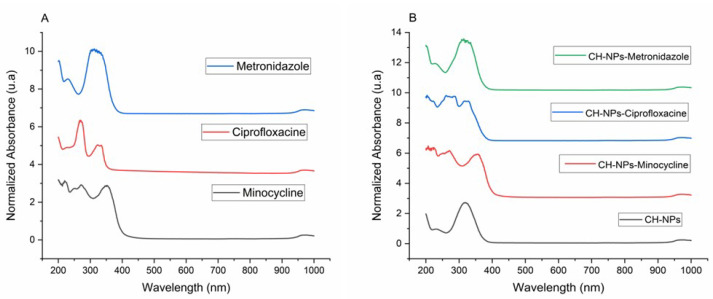
(**A**) UV-Vis spectra of Antibiotics, (**B**) UV-Vis spectra of CH-NPs and CH-NPs loaded with antibiotics.

UV-Vis analysis is based on the maximum ultraviolet absorbance of antibiotics and chitosan solutions with antibiotics, finding a wavelength for minocycline of 225 nm, 275 nm and 380 nm; for ciprofloxacin of 277 nm and for metronidazole of 270 nm (Figure 2A); for CH-NPs of 208 nm; CH-NPs-Minocycline 255 nm and 370 nm; CH-NPs-Ciprofloxacin 266 nm and 275 nm; CH-NPs-Metronidazole 220 nm and 277 nm using water as solvent (Figure 2B).

### 3.2. Agar Diffusion Assay

**Figure 3:**
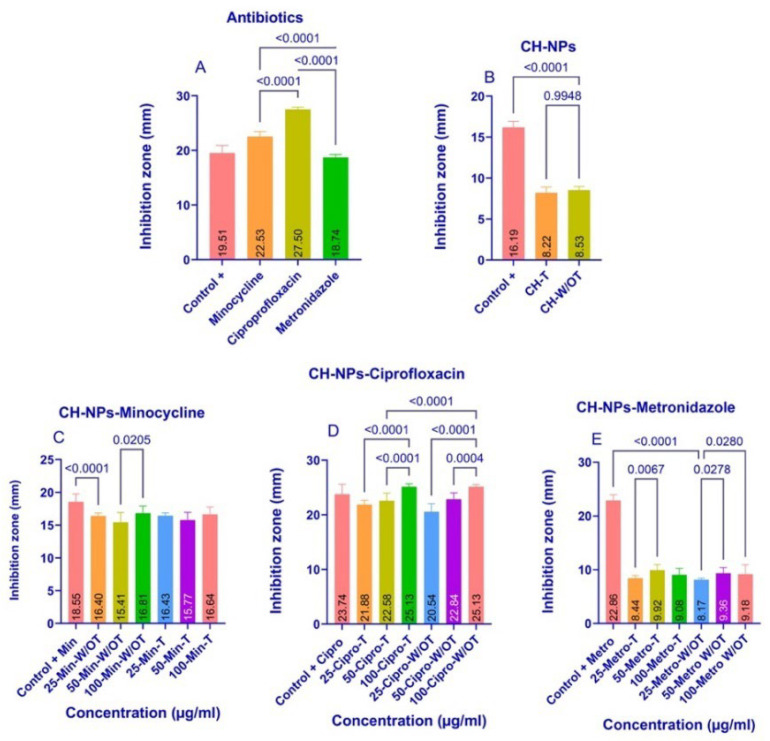
Bacterial Inhibition in the agar diffusion assay with *E. faecalis*, the average diameter of the inhibition zones of each experimental group, indicates the statistically significant differences with a *p* ≤ 0.05 value; (**A**) Antibiotics, (**B**) CH-NPs, (**C**) CH-NPs-Minocycline, (**D**) CH-NPs-Ciprofloxacin, (**E**) CH-NPs-Metronidazole.

The results of the agar diffusion assay with *E. faecalis* are shown in Figure 3A–E, which displays the mean values of the inhibition zones. The study groups that showed statistically significant differences in the analysis of variance (ANOVA) are identified using Tukey’s multiple comparisons test (*p* ≤ 0.05). The analysis was performed using Prism 10 for Windows, version 10.2.3 (2024). The greatest differences were observed in the antibiotic group (Figure 3A), with ciprofloxacin exhibiting greater antibacterial inhibition than minocycline (*p* = 0.001) and metronidazole (*p* = 0.001). CH-NPs demonstrated good antibacterial activity against *E. faecalis* (Figure 3B). The synthesis of these nanoparticles revealed statistically significant differences (*p* = 0.9948) in the increase in antibacterial inhibition when the temperature was increased during synthesis. However, despite their inhibitory effect, they did not show greater antibacterial inhibition than the positive control (CHx).

Figure 3C shows that the control group with CHx exhibited greater bacterial inhibition than the 25-CH-NPs-Min W/OT group (*p* = 0.0001), and that the 100-CH-NPs-Min W/OT group showed greater bacterial inhibition than the other experimental W/OT groups (*p* = 0.0205). However, despite these differences, all groups demonstrated a good ability to inhibit the growth of *E. faecalis*.

Figure 3D shows that samples 100-CH-NPs-Cipro-T and W/OT exhibited greater bacterial inhibition than samples 25-CH-NPs-Cipro-T and W/OT (*p* = 0.0001) and (*p* = 0.0001). Furthermore, all samples showed greater bacterial inhibition than the control group (+CHx).

Figure 3E shows that the 50-CH-NPs-Metro-T and W/OT concentrations exhibited greater bacterial inhibition against *E. faecalis* than the other concentrations tested, with *p*-values of 0.0067 and 0.0278, respectively. None of the CH-NPs-Metro W/T and W/OT were superior; however, the control +CHx group showed greater inhibition than the other ratios tested.

### 3.3. Microdilution Assay

**Figure 4:**
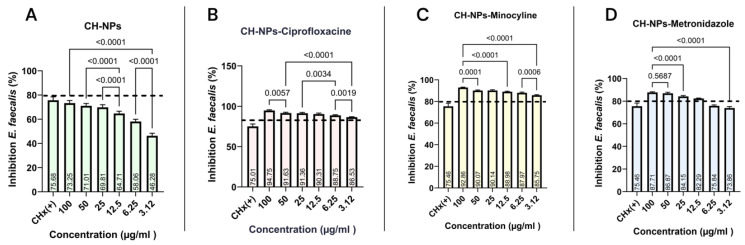
Bacterial inhibition of the microdilution assay with *E. faecalis*, with the average minimum inhibitory concentration value of each experimental group: (**A**) CH-NPs, (**B**) CH-NPs-Ciprofloxacin, (**C**) CH-NPs-Minocycline, (**D**) CH-NPs-Metronidazole. The dotted line indicates the reference point for greater than 80% bacterial inhibition.

To determine the MIC, the antibacterial activity against *E. faecalis* was evaluated over a 24 h period. Figure 4A–D shows the results obtained with the CH-NPs solution in combination with each of the antibiotics. Figure 4A shows that the CH-NPs are effective against *E. faecalis*; however, bacterial inhibition exceeding 80% was not observed at any concentration. The highest inhibition was observed at a concentration of 100 μg/mL, with 73.25%, which was greater than that observed at the other concentrations tested.

Chitosan nanoparticles loaded with ciprofloxacin (Figure 4B) showed excellent antibacterial activity against *E. faecalis*, with inhibition rates ranging from 94.75% at 100 μg/mL to 86.53% at 3.12 μg/mL. While significant differences were observed between the highest and lowest concentrations (*p* = 0.0001), all experimental groups exhibited higher antibacterial activity than the control group (CHx). Similarly, chitosan nanoparticles loaded with minocycline (Figure 4C) and metronidazole (Figure 4D) demonstrated the best antibacterial activity against *E. faecalis* at concentrations ranging from 100 μg/mL to 12.5 μg/mL.

### 3.4. Cytotoxicity Assay

**Figure 5:**
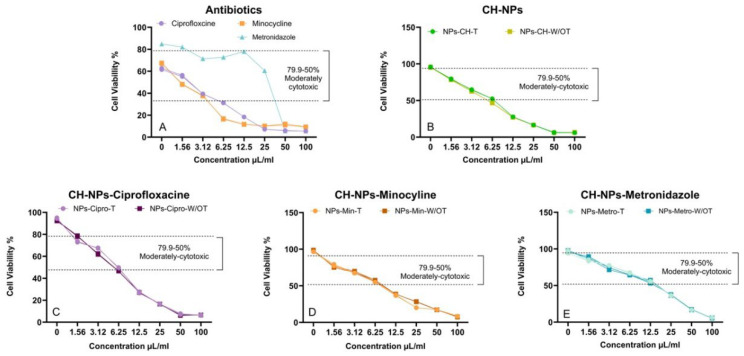
Cell viability response of CH-NPs with antibiotics in hDPSCs at a concentration of 5 × 10^4^ cells/mL. (**A**) CC_50_ of Antibiotics, (**B**) CC_50_ of CH-NPs, (**C**) CC_50_ of CH-NPs-Ciprofloxacine. (**D**) CC_50_ of CHNPs-Minocycline, (**E**) CC_50_ of CHNPs-Metronidazole.

Concentrations ranging from 0 to 100% (from 100 μg/mL to 1.56 μg/mL) were tested, and the proliferation of hDPSCs was analyzed after 24 h. The median cytotoxic concentration (CC_50_) was calculated, reporting that the CC_50_ of the antibiotics (Figure 5A).

For ciprofloxacin and minocycline, the concentration is 3.12 μg/mL, while for metronidazole, it ranges between 12.5 μg/mL and 25 μg/mL. In the case of CH-NPs with and without heat treatment (Figure 5B), the concentration is 6.25 μg/mL. The same applies to the CH-NPs-min (Figure 5C) and CH-NPs-cipro (Figure 5D). For the CH-NPs-metro, the concentration is 12.5 µg/mL. However, no differences in CC_50_ were observed when the temperature was varied during the synthesis of the antibiotic-loaded CH-NPs.

## 4. Discussion

### 4.1. Characterization of the CH-NPs

Tkachenko Y et al., 2022 state that FTIR is a useful analytical tool, used as a qualitative method to obtain information about vibrational non-linearity, spectral diffusion, and chemical exchange [46]. In this study, we used FTIR to observe and analyze the potential interaction between chitosan and antibiotics. Korniienko et al., 2024 [47] indicated that chitosan can bind to other organic structures due to the regular distribution of its NH_2_ groups. When mixed with other compounds, it undergoes a displacement reaction involving its hydrogen bonds. When analyzing CH-NPs in combination with antibiotics, it is likely that the compounds undergo bond disruption upon mixing, as molecules with three or more atoms can vibrate, stretch, or bend at specific energy levels, as noted by Trivedi Mahendra and Ma D [45,46]. However, despite the similarity between the spectra of CH and the antibiotics, these compounds retain their characteristic peaks; in Figure 1B, the NH_2_ groups at 1568 cm^−1^ and 1388 cm^−1^ are observed, which can be attributed to both CH and minocycline as reported by Wu et al., 2018 [48]. In addition to ciprofloxacin, Figure 1B shows the fluoroquinolone ring (C-F) at 1037 cm^−1^ after its mixture with CH as reported by Hibbard et al. (2023) [49]; and for metronidazole, the sulfoxide group (S=O) peak is observed at 1577 cm^−1^ as reported by Golj et al., 2025 [50] (Figure 1B). However, it is recommended to perform nuclear magnetic resonance, as suggested by Ma D et al., for a more precise analysis of molecular properties from a quantum-mechanical perspective [51]. This study was not conducted, as the research focused on the antibacterial effect and cytotoxicity of CH-NPs in combination with antibiotics; nevertheless, this aspect represents a potential avenue for future research.

### 4.2. Inhibition of Growth

Currently, there is a constant evolution in in vitro research, with increasingly sophisticated methods for performing microbiological diagnostic tests and inhibiting bacterial growth or causing bacterial death through the application of antibacterial agents. De Almeida et al. reported in 2020 that *E. faecalis* has a prevalence of 90% in cases of endodontic failure, demonstrating high resistance to antibacterial agents such as 6.5% sodium hypochlorite (NaOCl) and 2% chlorhexidine (CHx) [52]. However, a concentration between 2% and 2.5% NaOCl has been recommended due to its antimicrobial efficacy against *E. faecalis*. However, this concentration is still concerning since in clinical settings it can cause corrosive damage to endodontic instruments, as well as ulceration and necrosis of soft tissues, as reported by Hung Kiong W et al., 2023 [53]. This study performed a microdilution assay (Figure 4) and reported a 75% inhibition of *E. faecalis* by chlorhexidine, indicating its ineffectiveness due to the bacterium’s ability to impede the penetration of some antibacterial agents, adhere to inorganic surfaces, and invade dentinal tubules, as described by Rayos-Verdugo JY et al. [12]. Given its high pathogenicity, it is essential to develop new, effective treatment strategies. Palasuk et al. (2014) found that ciprofloxacin is the most effective antibiotic for treating *E. faecalis*, compared to minocycline and metronidazole [54]. The results of the agar diffusion assay are consistent with the findings described previously (Figure 3A). Metronidazole is effective against anaerobic and Gram-positive bacteria; however, this study showed a reduced antibacterial effect against *E. faecalis* in the agar diffusion assay (Figure 3E), a result similar to that reported by Chamorro-Petronacci et al. in 2022, who investigated its antibacterial effect when incorporated into mesoporous silica [55]. Nevertheless, the microdilution assay demonstrated bacterial inhibition of *E. faecalis* (Figure 3D) when the CH-NPs contained metronidazole, underscoring the importance of using both conventional phenotypic and molecular assay methods, as highlighted in a study by Salam MA et al. in 2023 [56]. These methods are considered the gold standard in microbiological assays for comparing antibacterial effects, as they provide both qualitative and quantitative data on bacterial activity. According to Khan et al. (2019) [57], the microdilution assay offers advantages over the agar diffusion assay, such as a higher degree of automation, a lower margin of error, and a reduced risk of contamination, allowing for an objective and quantitative assessment of bacterial inhibition. Comparing the antibacterial effects of CH-NPs with those of antibiotics using microdilution and agar diffusion assays reveals more significant differences, which is the focus of this study. These practical implications could have a significant impact on the use of antibiotics in microbiology.

This research has demonstrated that CH-NPs are effective when loaded with antibiotics. The most effective antibacterial effect was observed when CH-NPs were loaded with ciprofloxacin, demonstrating a synergistic effect, as also observed in a study by Chamorro-Petronacci et al. in 2022 [55]. A recent study by Pascale et al. (2023) and Belkadi R et al. (2024) concluded that irrigation with 0.2% chitosan is as effective as 5.25% sodium hypochlorite solution in reducing the bacterial load of *E. faecalis*, supporting the efficacy of CH-NPs loaded with antibiotics, as shown in this study [58,59]. Korniienko V et al. (2024) report the properties of CH in medicine, widely recognizing its properties in the field of biomaterials for its excellent biocompatibility, biodegradability, inherent antibacterial properties and anti-inflammatory capabilities [47]. However, it is crucial to consider that endodontic biofilms are polybacterial, so further in vitro studies are needed to evaluate the antibacterial effect against the different bacteria present in the root canal microbiota. Regarding the influence of temperature during the synthesis of CH-NPs loaded with antibiotics, no study has reported significant differences in bacterial inhibition, as demonstrated in this study.

### 4.3. Cytotoxicity Assay

The significance of performing in vitro cytotoxicity assays stems from the necessity to ascertain whether a substance poses potential risks, especially in relation to the drugs examined within this study, and to facilitate the development of safer and more efficacious therapeutic strategies and medications. The combination of metronidazole, ciprofloxacin, and minocycline has been shown to be effective when administered via the intraductal route (Junior JF et al.). These antibiotics can sterilize the ducts, and therefore, they have been investigated both individually and in combination with biodegradable biopolymers [6]. Ahmed TA and Aljaeid BM reported that chitosan is the second most abundant natural polysaccharide on Earth and that, when mixed with certain drugs, it allows the formation of electrospun structures, thus opening the possibility of creating a new drug delivery system [60]. In the cytotoxicity assay performed in this study, ciprofloxacin and minocycline showed a CC_50_ of 3.12 µg/mL, while metronidazole demonstrated cell viability between 12.5 and 25 µg/mL in human adipose-derived stem cells (hADSC) (Figure 5A). This finding is consistent with the results of Grierosu C et al., who verified the moderate cytotoxicity of this antibiotic by analyzing its effect on cell viability [61]. The cytotoxic concentration 50 (CC_50_) of the CH-NPs containing the antibiotic indicated its effectiveness at concentrations ranging from 6.25 μg/mL to 12.5 μg/mL (Figure 5C–E). Therefore, it was possible to confirm its biocompatibility, demonstrating that low doses are effective on hDPSCs. Vouzara T et al. (2016) reported that endodontic irrigants exhibit dose- and time-dependent cytotoxicity, with results similar to those obtained when the dose plays a crucial role in this assay [62]. The utilization of endodontic irrigants combined with antibiotic or antiseptic solutions stems from the necessity to develop novel disinfection strategies capable of addressing the limitations inherent in mechanical instrumentation alone. NaOCl is predominantly used due to its ability to dissolve pulp tissue and other organic constituents at concentrations of 2% to 2.5%. Nevertheless, its cytotoxic effects remain a concern, as reported by Hung et al. (2023), since accidental extrusion may lead to necrosis of adjacent tissues. Additionally, alternating irrigation with NaOCl and CHx can result in the formation of a brown precipitate that may contain parachloroaniline, a byproduct with mutagenic potential [53]. Pascale et al., 2023 highlighted chitosan for its use in endodontics due to its antibacterial, tissue regeneration, and chelating properties [58], and Khan et al., 2019 demonstrated that chitosan treatment improves the mechanical and structural properties of dentin because its use as a biological nanocarrier provides additional advantages in its diffusion through the endodontic biofilm [57]. Currently, several strategies have been proposed to improve irrigation systems by applying nanoparticles, providing therapeutic efficacy with minimal side effects. Belkadi R. et al. in 2024, reported the effect of CH-NPs in a 0.2% irrigant solution, comparing them with other substances for endodontic use, evaluating their cell viability in stem cells of the apical papilla, demonstrating that CH-NPs were less cytotoxic than the rest of the irrigants, suggesting the application of new alternatives to the disinfection protocol through the use of irrigants with lower cytotoxic properties taking into account the concentration and exposure time [59]. Alghofail M et al. (2024) analyzed the viability and proliferation of human mesenchymal stem cells (hMSCs) using an Alamar Blue assay on chitosan-gelatin (CH-G) scaffolds loaded with a slow-release antibiotic formulation (Augmentin; 0.1 mg/mL), commonly used in regenerative endodontic procedures, and staining methods. They observed good cell viability. In this study, they demonstrated important cell phenotypes, including the multipotency and differentiation capacity of hMSCs, which are comparable to those of hDPSCs used in the present study [24]. Regarding the influence of temperature on the synthesis of CH-NPs containing antibiotics, no study to date has reported statistically significant differences in cytotoxicity increases in hDPSC cells, as demonstrated in this study. This underscores the importance of employing innovative organic materials in endodontics that possess suitable properties for direct contact with dental or periapical tissues, while also ensuring their biocompatibility and biodegradability.

## 5. Conclusions

Chitosan has become one of the most promising biodegradable biopolymers due to its diverse properties, such as moderate to low cytotoxicity, notable antibacterial activity, good biocompatibility, and drug carrier capabilities. Its use as an endodontic irrigant combined with broad-spectrum antibiotics has gained increasing interest because of its potential to enhance antibacterial effectiveness and biocompatibility, paving the way for new therapeutic strategies in this area. The CH-NPs-ciprofloxacin showed the strongest antibacterial activity against *E. faecalis* in both agar diffusion and microdilution tests, making it the top choice among nanoparticles for targeting this bacterium. CH-NPs-metronidazole effectively suppressed *E. faecalis* in microdilution assays and also had lower cytotoxicity on hDPSCs. Conversely, CH-NPs-minocycline demonstrated better antibacterial effects than plain chitosan in microdilution tests, though it exhibited higher cytotoxicity; the agar diffusion results were less promising. Changes in synthesis temperature did not significantly affect the antibacterial activity or cytotoxicity of the CH-NPs, indicating the need for further research to optimize their use in endodontics. This study provides important insights for endodontic applications and is a vital step for future investigations. Nonetheless, this field remains evolving and relatively underexplored. Reassessing the use of irrigation solutions is crucial, given the limited evidence on chitosan nanoparticles combined with antibiotics.

## Data Availability

The raw data supporting the conclusions of this article will be made available by the authors on request.

## Abbreviations

The following abbreviations are used in this manuscript:

CH-NPs	Chitosan nanoparticles
FTIR	Fourier Transform Infrared Spectroscopy
CHx	Chlorhexidine
*E. faecalis*	*Enteroccocus faecalis*
hDPSC	Human dental pulp stem cells
ENES	National School of Higher Studies
W/OT	Room temperature
W/T	Temperature
MIC	Minimum inhibitory concentration
MTT	Thiazolyl blue tetrazolium bromide
DMSO	Dimethyl sulfoxide
MEM	Minimum essential medium
CC50	Average cell cytotoxicity value
CH-NPs-Min-T	Chitosan nanoparticles with minocycline with temperature
CH-NPs-Min-W/OT	Chitosan nanoparticles with minocyclin, room temperature
CH-NPs-Cipro-T	Chitosan nanoparticles with ciprofloxacine with temperature
CH-NPs-Cipro-W/OT	Chitosan nanoparticles with ciprofloxacine, room temperature
CH-NPs-Metro-T	Chitosan nanoparticles with metronidazole with temperature
CH-NPs-Metro-W/OT	Chitosan nanoparticles with metronidazole, room temperature
